# Modeling Endometrial Cancer: Past, Present, and Future

**DOI:** 10.3390/ijms19082348

**Published:** 2018-08-09

**Authors:** Tom Van Nyen, Cristian P. Moiola, Eva Colas, Daniela Annibali, Frédéric Amant

**Affiliations:** 1 Department of Oncology, Gynecological Oncology, KU Leuven, 3000 Leuven, Belgium; tom.vannyen@kuleuven.be (T.V.N.); daniela.annibali@kuleuven.be (D.A.); 2 Pathological Oncology Group, Biomedical Research Institute of Lleida (IRBLLEIDA), University Hospital Arnau de Vilanova, 25198 Lleida, Spain; cristian.pablo@vhir.org; 3 Biomedical Research Group in Gynecology, Vall Hebron Institute of Research, CIBERONC, 08035 Barcelona, Spain; eva.colas@vhir.org; 4 Centre for Gynecologic Oncology Amsterdam (CGOA), Antoni Van Leeuwenhoek-Netherlands Cancer Institute (Avl-NKI) and University Medical Centra (UMC), 1066 CX Amsterdam, The Netherlands

**Keywords:** endometrial cancer, preclinical models, translational research

## Abstract

Endometrial cancer is the most common type of cancer of the female reproductive tract. Although prognosis is generally good for patients with low-grade and early-stage diseases, the outcomes for high-grade and metastatic/recurrent cases remain poor, since traditional chemotherapy regimens based on platinum and taxanes have limited effects. No targeted agents have been approved so far, although several new drugs have been tested without striking results in clinical trials. Over the last decades, many efforts have been made towards the establishment and development of preclinical models, aiming at recapitulating the structural and molecular determinants of the disease. Here, we present an overview of the most commonly used in vitro and in vivo models and discuss their peculiar features, describing their main applications and the value in the advancement of both fundamental and translational endometrial cancer research.

## 1. Introduction

Endometrial cancer (EC) accounts for 4.8% of all cancers diagnosed in women and is the fifth most common type of cancer in developed countries [1,2,3]. It is the most common malignancy of the female reproductive tract, with a cumulative risk of 1% by age of 75 years, while the death risk is 0.2% [1,2,3]. Around 320,000 new cases are diagnosed yearly worldwide, and 76,000 patients die every year from the disease [1,2,3]. Around 75% of all ECs are diagnosed as FIGO (International Federation of Gynecology and Obstetrics) stage I or II, corresponding with a 5-year overall survival that varies between 74% and 91%. Patients diagnosed as FIGO stage III or IV have a 5-year overall survival rate of 57–65% and 20–26%, respectively [3,4,5].

Recognized risk factors for the development of EC are (i) exposure to unopposed estrogens or to tamoxifen, (ii) diabetes, (iii) obesity, (iv) nulliparity, (v) early-onset menarche, and (vi) late-onset menopause, amongst others [6]. The increasing aging of the population together with higher frequencies of metabolic diseases and diabetes are possible explanations for the observed higher incidence rates of EC in the developed world (i.e., Western Europe and Northern America), compared to other regions [3]. Patients typically present with abnormal uterine bleeding and, in case of advanced disease, possibly abdominal and pelvic pain [7]. Standard diagnostic procedures consist of pelvic ultrasonography, endometrial biopsy, and hysteroscopy when the diagnosis is uncertain [3]. Treatment is primarily based on cytoreductive surgery, mostly total hysterectomy and salpingo-oophorectomy [3]. Depending on different prognostic factors such as tumor grade, histology, and myometrial and cervical invasion, patients can be divided into low-risk or high-risk groups, related to a long and short disease-free survival, respectively [8]. For high-risk patients and those with metastatic disease, surgery is combined with adjuvant radiotherapy and/or chemotherapy such as cisplatin, carboplatin, doxorubicin, and cyclophosphamide [6,8,9]. Since for recurrent and metastatic disease only limited treatment options are available [10,11,12,13], the last decade saw growing interest in novel targeted therapies.

Traditionally, EC has been divided into two subtypes with distinct clinical, pathological, histological and molecular behavior [14,15]. Type I EC are mainly low grade, estrogen-dependent, hormone-receptor-positive adenocarcinomas with endometrioid morphology and are often referred to as endometrioid endometrial cancers (EECs). EECs account for 85% of all ECs. Moreover, they are mostly diagnosed at an early stage and are generally characterized by a good prognosis [3,16]. Type I tumors often show alterations in the PI3K/Akt pathway, suggesting they could potentially respond to anti-PI3K/Akt therapies [13]. Commonly mutated genes include phosphatidylinositol-4,5-bisphosphate 3-kinase catalytic subunit alpha (PIK3CA), KRAS proto-oncogene, GTPase (KRAS), fibroblast growth factor receptor 2 (FGFR2), and Catenin beta 1 (CTNNB1), amongst others [17]. The most frequently altered gene is the tumor suppressor Phosphatase and tensin homolog (PTEN), in approximately 50% of all cases, while the most common altered oncogene is KRAS, altered in 25% of cases [18]. Type II ECs are characterized by non-endometrioid histology and include carcinosarcomas, serous and clear cell carcinomas, and all tumors with different histology and molecular features [16]. Type II ECs are generally high grade, hormone-receptor negative, and have poor survival rates [3]. Serous carcinomas only account for 10% of all ECs, although they are responsible for 39% of the total EC deaths [19]. The overall survival rate for serous carcinoma and clear cell carcinoma is only 56% compared to the 86% reported for endometrioid carcinoma [20]. Type II ECs are characterized by high frequencies of Tumor protein p53 (TP53) mutations and other low-frequency genomic alterations, such as F-box and WD repeat domain containing protein 7 (FBXW7) and AT-rich interaction domain 1A (ARID1A) mutations and Erb-b2 receptor tyrosine kinase 2 (ERBB2) amplification [17].

The Cancer Genome Atlas recently identified four distinct EC molecular subtypes, i.e., the Polymerase e (POLE) ultramutated, the microsatellite instability hypermutated, the copy-number low microsatellite stable, and the copy-number high serous-like subgroups [17]. These subtypes show increasing grade, TP53 mutations, and somatic copy number alterations as well as decreasing mutation rates, respectively [17]. However, around 40% of all ECs belongs to a large nonspecific molecular profile (NSMP) subgroup, characterized by the absence of POLE or TP53 mutations and microsatellite instability. Recently, a somatic copy-number alterations (SCNA) analysis identified a different subgroup within the NSMP EC, refining the molecular classification of these poorly-characterized tumors. The proposed subgroup shows amplifications of 1q32.1, the locus where Double minute 4 protein (MDM4) is located, and, importantly, this type of amplification has been identified as a prognostic marker [21].

**Figure 1:**
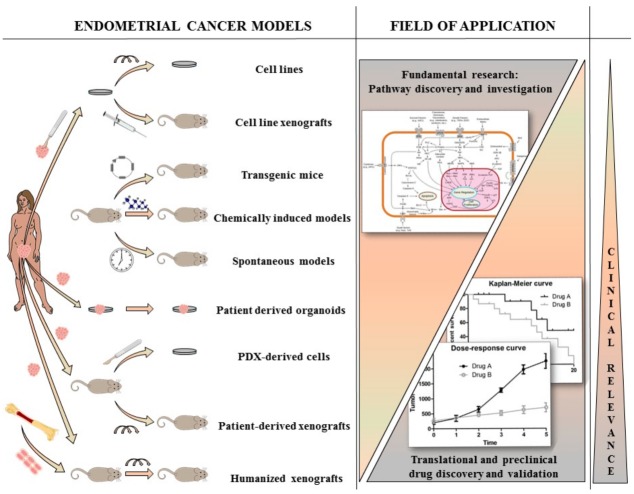
Presentation of the different available preclinical models present for endometrial cancer (EC) research. Cell lines and their derived xenografts are models for basic EC research however lack clinical relevance. Better, in vivo models are transgenic, chemically induced and spontaneous models, however they lack patient-derived properties. Patient-derived models (organoids, xenografts and humanized mice) have the highest clinical relevance and are useful for translational and preclinical drug discovery and validation, however they are less likely to be used for fundamental research.

Our knowledge about EC biology has been increased during the past decades and continues to grow thanks to the use of many different preclinical models. With this review, we aim to discuss the general aspects of the different preclinical models available, their peculiar features and refinements, how they have been used to study EC, which progresses they enabled in our understanding of the disease, and their future challenges and applications, as highlighted in the graphical abstract (Figure 1).

## 2. Cell Lines and Cell Line-Derived Xenograft Models

Historically, in vitro cell lines have had a prominent role in anticancer drug development [22], although such models may lack clinical relevance due to the immortalization and adaptation processes induced by the continuous growth on plastic. The possibility of working under standardized conditions makes cell lines extremely useful for the discovery of molecular mechanisms and biological pathways related to an observed phenotype, while also allowing for cost-effective high-throughput screenings. However, it is worth noting that they are exposed to altered oxygen levels and nutrient composition, compared to the original tumors grown in the patients, and that they lack any sort of interaction with the microenvironment [22].

For endometrial cancer, multiple different cell lines have been established during the last decades (Table 1). The most commonly used cell lines—AN3CA, ECC-1, HEC1A, HEC1B, and Ishikawa—are type I tumor-derived cell lines, harboring alterations in the PI3K/Akt pathway, thereby representing the majority of EC tumors in the clinic. Short tandem repeat (STR) profiling of 10 of the most common EC cell lines showed that HEC1A, HEC1B, HEC50, AN3CA, KLE, and RL-95-2 have unique STR profiles, consistent with their originally derived tumors. Different variants of Ishikawa cell lines showed polymorphic genomic regions; however, high similarity profiles indicate that they originate from the same patient. Korch and colleagues genotyped different cell lines and found that the ECC-1 cell line does not match the original EnCa-101 tumor [23]. Therefore, the ECC-1 cell line has been discontinued and is no longer commercially available. This example points to the importance of proper annotation and to the need of a standardized authentication system for cell lines.

Tumor cells isolated from one single patient can lead to different cell lines, as illustrated by the HEC1A and HEC1B cell lines derived from the same surgical specimen [24], which differ in their microsatellite instability phenotype. This observation highlights that most of the info related to intra-tumor heterogeneity cannot be maintained in vitro by establishing only one cell line from one tumor. However, where available, paired cell lines originating from the same patient are extremely important to investigate this and other biological issues. Using the HECs cell lines, for example, Glaab and colleagues showed the significance of mismatch repair endonuclease PMS2 (PMS2) in the maintenance of genomic stability in human cells [24].

Nevertheless, peculiar molecular aberrations have been shown to be maintained when passaging a tumor in vitro. Specifically, alterations in the PI3K/Akt pathway have been observed in both EEC patients’ tumors and EEC cell lines. Weigelt et al. analyzed 24 commercial EEC cell lines and described mutations in PTEN, PIK3CA, PIK3R1, and KRAS [25], with frequencies comparable to those seen in human EEC samples [25,26,27,28]. An analysis of gene copy number aberrations in the five most common EC cell lines—Ishikawa, HEC1A, HEC1B, EEC-1, and AN3CA—showed that the PI3K/Akt and Wnt pathways are commonly affected [29]. Furthermore, the PI3K/Akt/mTOR pathway has been validated as a potential target for novel targeted therapies [25], and Philip and colleagues recently showed that a combination of PI3K and Poly (ADP-ribose) polymerase (PARP) inhibition has synergistic effects in PTEN mutated cells [30]. Using EC cell lines, Lin et al. showed that cisplatin, which is a main therapeutic agent, exerts its effect by regulating autophagy through the PI3K/Akt pathway and that PI3K/Akt inducers could reverse cisplatin activity [31].

Chemoresistance and metastatic dissemination remain major hurdles for EC patients, and different chemotherapy resistance mechanisms have been described [12,32]. Recently, it has been shown that non-coding RNAs (e.g., of miR-139-5p and miR-143) might play a role in tumor growth, therapy resistance, and metastasis [33]. A different report indicated that the long non-coding RNA homeobox transcript antisense RNA (HOTAIR) contributes to platinum resistance in vitro [34] and that miR-205 is able to inhibit cell growth in progesterone-resistant Ishikawa cells [35].

Other fields of interest that make use of EC cell lines, often as starting models, are summarized in Table 2.

The use of large-scale omics technologies revealed marked intra- and inter-tumor heterogeneity in patients, which cannot be captured by single cell lines. Therefore, nowadays large cell line panels are often used to try to recapitulate as much as possible in vitro tumor heterogeneity and to identify genomic determinants of drug sensitivity. The most known panels are the National Cancer Institute 60 (NCI60) platform [54] and the Japanese Foundation for Cancer Research 39 (JFCR-39) [55], which do not, however, list EC lines, as well as the Center for Molecular Therapeutics 1000 (CMT1000), where ECs are represented by different cell lines [22,56].

Since the information about cell lines established decades ago is often scattered and lacks systematic annotations, the Broad Institute launched the Cancer Cell Line Encyclopedia (CCLE) initiative in 2012, with the aim of compiling genomic datasets and pharmacological response profiles of different cancer cell lines to selected compounds. Today, the CCLE counts more than 13,000 unique datasets (for gene expression, chromosomal copy number analyses, and mutational sequencing) from 1457 cell lines, of which 28 are ECs.

## 3. Organoids and Organs-on-a-Chip Models

The high attrition rates observed for novel compounds in oncology, due to the discrepancies between results obtained in preclinical and clinical settings, has been for long imputed to the use of suboptimal models, lacking predicting value in terms of therapeutic response. Two important factors that strongly limit the clinical relevance of the conventionally used cancer cell lines are the lack of interaction with the stromal compartment and the scarcity of normal tissue-derived counterparts. For this reason, significant efforts have been spent in the last decade to develop new ex vivo models that would better mimic the original tumors’ physiology.

### 3.1. EC Organoids Models

The discovery that both healthy and tumor tissues can grow in vitro as self-organizing three-dimensional (3D) structures under specific growth conditions opened new perspectives for organoid cultures. Organoid models have been established from healthy human or mouse endometrium by Boretto et al. [57], as well as from endometrial cancer patients by Turco and colleagues, who adapted conditions used to grow adult human stem cells-derived organoids [58]. EC organoids maintained the architecture of the original tumors under a chemically defined medium, could be grown for extended periods of time (5 months), and showed genetic and molecular stability. Since the healthy endometrium is a dynamic and plastic tissue adapting and regenerating in response to hormonal cycles, the role of stem/progenitor cells is also being investigated during malignant transformation. Interestingly, it has been shown that endometrium progenitor cells display a high capacity to differentiate into cytokeratin-positive organoid cultures [59] and that cells highly expressing ALDH (alcohol dehydrogenase), a stemness marker for the endometrium, have a high organoid forming capacity [60].

EC patient-derived spheroids have been recently used to perform a pharmacological screening with 79 different targeted therapies by Kiyohara and colleagues, which showed that non-endometrioid carcinomas seem to be highly sensitive to survivin inhibition, while endometrioid cancers could be resistant [61]. Girda et al. established 15 patient-derived organoid cultures on which they screened multiple drugs. They observed that STAT3 inhibition does impede organoid formation in almost all cultures, confirming the key role of cancer stem cells in tumor growth and organoids establishment. Surprisingly, none of the cultures was affected by cisplatin or by different progestins. On the other hand, strong growth inhibition was observed in paclitaxel-treated organoid cultures, while moderate inhibition was described for tyrosine kinase inhibitors and fulvestrant treatment [62]. Because they are relatively fast to establish and easy to maintain in culture, organoids have also be used to provide proof of concept for drug repurposing in EC, as reported by Dasari et al. who found that Verteporfin, a photosensitizer normally used for photodynamic therapy in conditions such as macular degeneration, could potentially be effective in EC [63].

### 3.2. Organs-on-Chip Models

The so-called organs-on-chip (OOC) models are microfluidics systems where engineered biomimetic chambers containing cells or tissues are connected and continuously perfused by circulating medium, so as to simulate the physiological dynamics and functionality of tissues within one organ or the crosstalk between different organs [64].

Applied to the investigation of cancer tissues, such microfluidics technologies have high potential to become the future gold standard for drug testing in translational research. The approach to growing patient-derived tumor cells or tissues on chips proved to be feasible for lung and breast cancers and was shown to be capable of mimicking tumor growth, dormancy, invasion, and response to therapy [65,66]. In this view, the recent development of a multi-organ microfluidic system, called EVATAR, which simulates the human female reproductive tract and recapitulates its hormonally-controlled dynamics, paves the way for future applications related to gynecological pathologies, spanning from endometriosis to ovarian, cervical, and endometrial cancers [67].

## 4. In Vivo Models

Mouse and rat models are appreciated as standard animal models in translational cancer research, mainly because they are easily available and allow drug testing on a population scale, due to the short time needed to generate results, with the advantage of having tumor cells growing as 3D masses and in strict connection with the stromal compartment. However, they are also subject to caveats, when it comes to comparing the biology of murine and human tumors. A first point to consider is that humans live around 30–50 times longer compared to mice and rats, are ~3000 times larger, and therefore undergo much more cell divisions. This implies that tumors might develop and evolve differently. It was indeed shown that rodent cells require at least two genetic alterations before gaining tumorigenic potential, while human cells are more difficult to transform [68]. Interestingly, the lifetime risk for developing cancer is comparable in rodents and humans. More specifically, 30% of laboratory rodents develop cancer by the end of their lifetime and 30% of humans develop cancer by 70–80 years of age [69]. However, it is known that at least some of the antineoplastic mechanisms that have been described (e.g., limited replicative potential and growth signaling self-insufficiency) are human-specific, implying possible differences in response to carcinogenesis and cancer chemopreventive agents [69,70]. Several excellent reviews are available on this subject [69,70,71].

### 4.1. Spontaneous EC Rodent Models

It is known that, just like humans, rodents can also spontaneously develop tumors if kept alive until their natural life end. However, what was surprising for researchers is that some specific rat strains have an abnormally high incidence rate of EC. In 1981, Deerberg and colleagues already noticed a 39% incidence rate of uterine tumors in female Han:Wistar rats [72,73].

Later, different spontaneous EC rat models were also described. Nagaoka et al. showed that in Donryu rats the incidence rate of endometrial adenocarcinoma was as high as 35.1% and that around 60% of all rats would develop proliferative lesions in the endometrium [74]. Tanoguchi et al. showed that KRAS mutation frequencies in tumors originating in Donryu rats are similar to those observed in human EC, suggesting potential relevance for the findings coming from this model [75].

Donryu rats have been historically used for the investigation of EC etiology and they helped to elucidate that hormonal imbalance, more specifically an increased estrogen:progesterone (E:P) ratio, can be linked to EC development [76,77]. Yoshida et al. and Kojima et al. showed that compounds decreasing the E:P ratio, such as bromocriptine and indole-3-carbinole, have a protective effect against EC [77,78], while compounds increasing the E:P ratio have the opposite effect. These observations explain why neonatal exposure to a high dose of the estrogenic compound p-t-octylphenol increases the likelihood of EC development [79]. Also, isoflavone aglycones, chemical compounds found in soy products that exhibit estrogen-like properties, have been shown to facilitate EC development in Donryu rats [80]. The notion that high-fat diets increase the E:P ratio, thus leading to an increased risk of EC incidence, came from studies in spontaneous rat models [81]. In addition, Nagaoka and colleagues showed that multiparity is linked with hormonal changes and leads to the suppression of EC incidence rate if compared to nulliparity in vivo [82].

Besides Donryu rats, other spontaneous EC rat models are available, such as DA/Han rats, BDII/Han rats, and the low spontaneous EC incidence rat strain F344. DA/Han rats are an inbred rat strain that exhibits high spontaneous tumor development (>60% if kept until the natural life end, 24–27 months) and has a high metastatic phenotype [73]. However, only limited information is present in the literature and these rats have not been widely used in EC research.

BDII/Han rats, on the other hand, are probably the better characterized spontaneous EC model, both at genomic and molecular levels. If kept to their natural life end (around 26–27 months), over 90% of the female rats die due to endometrial carcinoma [73,83]. Using gene sequencing and real-time PCR, Samuelson et al. showed that tumors in BDII/Han rats are molecularly similar to type I endometrial tumors. Amongst other alterations, they found allelic imbalance and altered expression of PTEN, and only limited aberrations in TP53 [84]. Of note, tumors in BDII/Han rats are hormone-sensitive, like type I human EC, since it has been shown that in rats ovariectomized prior to estrous cyclicity the tumor incidence rate decreased to 0% [73]. Another piece of evidence is that melengestrol acetate administration suppresses tumor growth [85]. Tumors from BDII/Han rats have been extensively genomically characterized, and the results suggest that the upregulation of CDK6 and/or Met could play a role in the development of cancerous lesions [86,87,88,89,90,91,92,93,94].

Recently, hormone receptor expression was evaluated in the Fischer 344 (F344) rat strain [95]. F344 is not a high-incidence EC rat strain; however, it is one of the most commonly used for carcinogenicity testing. Tumors originating in F344 display high inter- and intra-tumor heterogeneity in terms of estrogen receptor (ER) and progesterone receptor (PR) expression, as observed in women, with the majority being ER^+^PR^+^. Of note, ER^+^ tumors in F344 tend to be well differentiated, as reported in humans. Although in women EC tumors are mostly PR^+^, F344 rats also develop PR^−^ tumors [95].

In conclusion, spontaneous EC rat models are excellent tools recapitulating some of the molecular and genomic features observed in human tumors. However, their value is generally underappreciated and they only have minimal use for drug testing in the preclinical setting. One of the drawbacks is that the timing of tumor development is difficult to predict since it is not possible to know if and when the lesions will develop and a long period of time may pass before they start to grow [69].

### 4.2. Chemically Induced EC Rodent Models

Although different spontaneous rat models for EC are available, tumors are still often induced by treatment with chemical compounds, such as artificial estrogens, in rat and/or mice models. Traditionally, researchers mainly use two related compounds to induce EC tumors in vivo, i.e., *N*-methyl-*N*-nitrosourea (MNU) [96,97] or *N*-ethyl-*N*-nitro-*N*-nitrosoguanidine (ENNG) [98,99]. These are alkylating agents which cause mutagenic and carcinogenic effects by alkylating DNA, RNA, and proteins [100], often in combination with estrogens [99,101,102]. Takahashi et al. showed that ENNG combined with estradiol, estrone, estriol, 16β-hydroxyestrone, 16α-hydroxyestrone, and 17-epiestriol significantly induces endometrial adenocarcinoma tumor formation and progression in ICR (Institute of Cancer Research) outbred mice [99]. All of these metabolites belong to the 16α-hydroxylation pathway or the upstream 16β-hydroxylation pathway of estrogen metabolism, while metabolites belonging to the downstream 16β-hydroxylation pathway and the 2-, 4-hydroxylation pathway, such as 2-hydroxyestriol, 2-methoxyestradiol, 2-methoxyestriol, and 16-epiestriol, have only limited to no effect on the growth of endometrial carcinomas [99].

Chemically induced EC animal models have been used as translational models to investigate the effects of chemopreventive agents. Niwa et al. used them to study the effect of danazol on endometrial carcinogenesis [97], while others investigated the effect of tamoxifen [103] and dietary indole-3-carbinole [98] on endometrial adenocarcinoma growth. A major drawback, however, is that exposure to the chemical compounds could have detrimental effects on the metabolism of specific tissues and organs. This strongly limits their use, since it implies that the metabolism of a novel drug could be somehow altered in animals that were exposed to chemicals [71].

### 4.3. Transgenic Mouse Models

Transgenic mice are mostly used for investigating biological mechanisms related to cancer development [71]. For EC, approaches based on different transgenes successfully led to the establishment of several models.

#### 4.3.1. PTEN Knock-Out Mouse Models

Since PTEN is the most altered gene in EC, its knock-down has successfully led to the development of transgenic EC models [18]. Knock-out of one of two alleles (PTEN^+/−^) is sufficient to generate hyperplasia, which develop to carcinoma in 20% of all cases, by the age of 10 months [104], while PTEN^−/−^ homozygosity is embryonically lethal [73]. However, to study homozygous PTEN deletions in adult mice, different conditional systems have been recently developed, such as a tamoxifen-inducible transgenic system [105], an adenovirus-mediated Cre-lox system [106], or the isolation of PTEN^loxP/loxP^ cells from the uterus of adult mice, followed by gene inactivation and re-implantation [107]. It has been shown that PTEN inactivation per se is sufficient to rapidly induce endometrial carcinoma [105]. Since microsatellite instability is a highly frequent event in endometrioid EC, Wang et al. established a transgenic mouse system that harbors a homozygote MLH^−/−^ deletion next to the heterozygous PTEN loss (PTEN^+/−^) and showed an accelerated onset of endometrial carcinoma [18,108], confirming the role of microsatellite instability in EC. The importance of alterations in the PI3K/Akt pathway and microsatellite instability was also confirmed in vivo using transgenic mice [18].

Transgenic mice have also been used to study additional genes possibly related to EC development and their cooperation with each other or with PTEN. Contreras et al. showed that the inactivation of Serine/threonine kinase 11 (LKB1)—a master regulator of the Adenosine monophosphate-activated protein kinase (AMPK)-mTOR signaling—is sufficient to drive endometrial cancer development [109]. Cheng and colleagues also developed a model with a combined loss of PTEN and LKB1, with which they showed that loss of both genes leads to EEC and short survival, with a high dependency on the hyper-activated Akt pathway [106].

Another interesting approach is based on the establishment of primary cultures from the tumors developed in transgenic mice, as shown for the PTEN knock-out models [110,111].

#### 4.3.2. TP53 Knock-Out Models

TP53 mutations are found in advanced type I EC and TP53 is the most commonly altered gene in type II EC. Daikoku and colleagues showed that conditional TP53 deletion alone does not lead to EC development, while a combined conditional PTEN^−/−^TP53^−/−^ deletion led to shorter survival and exacerbated disease state compared to PTEN^−/−^ mice only, thereby confirming the importance of TP53 alterations in advanced type I EC [104]. Although type I EC is the most investigated subtype of EC because of its high incidence rate, type II EC is more aggressive and has a higher relative death rate [112]. Akbay et al. showed that the deletion of protection of telomeres protein 1A (POT1A)—a component of the shelterin complex stabilizing telomeres—combined with TP53 loss led to the development of type II-like EC in a mouse model by 9 months of age. In addition, it led to the insurgence of metastasis in 100% of the mice at 15 months. These results point to the importance of telomere instability and TP53 mutations in type II EC [112].

#### 4.3.3. The Mitogen Inducible Gene 6 (MIG-6) Knock-Out Model

A different EC model has been established by knocking-out the Mitogen Inducible Gene 6 (MIG-6) [113], the expression of which is known to be regulated by mitogens and stress stimuli. MIG-6 is an immediate early response gene and acts as a negative regulator of EGFR signaling. It is a known progesterone receptor-regulated gene, and this can partially explain why a low E:P ratio is linked to low EC incidence. Using uterus-specific MIG-6 null transgenic mouse models, it was shown that MIG-6 has an estrogen-dependent tumor suppressive function [113]. Furthermore, it was shown that MIG-6 expression inversely correlated with the phosphorylation of ERK1/2 [114]. The estrogen-dependency of EC tumors was examined in PTEN deleted mice, leading to the conclusion that EC tumorigenesis is independent of estrogen in PTEN^+/−^ mice [115] and the depletion of estrogen predominantly leads to neoplastic lesions, possibly explaining why endometrial carcinoma incidence is higher in peri- and postmenopausal women [116].

#### 4.3.4. Transgenic Models: Remarks

In many cases, transgenic mice are used to investigate response to therapeutic agents. Different Akt and mTOR inhibitors have been tested in transgenic mice, showing good responses [106,109]. Recent preclinical studies using transgenic EC mice tested olaparib (PARP-inhibitor) [107], dienogest (fourth-generation progestin) [117], and palbociclib (CDK4,6 inhibitor) [118]. Such models have also been used to evaluate the effect of diet on EC tumorigenesis, showing that the elevation of ω-3-polyunsaturated fatty acids attenuates PTEN deficiency-induced EC development [119].

However, important caveats must be considered when these previously established transgenic mice are used in preclinical studies. First, the genetic insertion copy number and insertion site in the genome are mostly unknown, but they can have a major influence on treatment response. Temporal aspects of transgene activation should also not be neglected [71]. Finally, transgenic tumors lack naturally occurring heterogeneity and in this sense are not fully representative of human tumors.

### 4.4. Patient-Derived Xenografts (PDXs) and Humanized Mice

Patient-derived xenograft models (PDXs) are established by implanting a piece of freshly isolated tumor from a patient directly into immunocompromised mice [120]. Tumor pieces can be implanted heterotopically or orthotopically [120]. The orthotopic implantation has several advantages because the tumor develops within the same anatomic environment as the original one in the patient. However, this kind of implantation is technically challenging and implies the need for imaging systems to monitor tumor growth, which is why heterotopic implantations are often used to generate PDX models. Subcutaneously accessible implantation sites include the flanks, the mammary fat pad, the interscapular fat pad, and the renal capsule [121]. Different mouse strains can be used; athymic nude mice, non-obese diabetic/severe combined immune deficiency (NOD/SCID) mice, and NOD/SCID/interleukin-2 receptor common γ-chain (IL2-Rγ)-deficient (NSG) mice can be chosen based on the desired degree of immunosuppression [120,121]. The engraftment success rate ranges depending on the tumor type, the used mouse strain, and the specific implantation method. However, in general, it has been observed that engraftment is more likely to occur for metastatic tissue compared to primary tumor tissue, and can reach up to 90% [121]. Multiple studies have shown that PDX models maintain the original histological, molecular, and functional heterogeneity present in the patients’ tumors over different cancer types [121,122,123]. What makes them an excellent model for translational cancer research is that PDXs can capture the complexity of the original human tumor (with high molecular and histological stability), they can predict the clinical response in patients [121,123,124,125], and they thus can be used as preclinical models for the validation of novel drugs and targeted therapies. PDXs can potentially be used for high-throughput drug screening [122,126]. In order to do so, Gao et al. showed the feasibility of the “one animal per model per treatment” (1 × 1 × 1) approach for drug screening [126]. Bruna et al. also showed that the use of PDX-derived short-term cell cultures (PDCs) are useful and are a better clinical model compared to conventional cell lines [122,126]. Furthermore, PDX models and their derived cells can be used for new biomarkers discovery and to investigate resistance mechanisms to treatments. They can potentially be used in xenopatient trials, co-clinical trials, and eventually in personalized medicine [123]. Recently, many efforts have been directed toward the development of humanized mice, in which a human immune system is (partially) restored, in order to investigate tumor interaction with the microenvironment and to investigate the role of the immune system in tumor growth and treatment response [127]. Although such systems are not available for EC yet, they might be interesting because immune blockade with immune checkpoint inhibitors are upcoming treatments for advanced and recurrent EC [128].

A detailed overview of the available EC PDX models and humanized mice can be found in the review article by Moiola et al. in this Special Issue [129].

## 5. Conclusions and Future Perspectives

During the last decades, our understanding of endometrial cancer biology increased mainly thanks to the advance of molecular techniques applied to the different available preclinical models.

Both in vitro and in vivo models helped to elucidate different aspects of the disease and paved the way for future preclinical and clinical investigations. Since a model is by definition imperfect in mimicking a real situation, and it naturally has concrete advantages for one aspect but disadvantages for another. Thus, the idea of employing integrative preclinical platforms with different models for one cancer type is gaining the interest of the scientific community. The exploitation of effective precision medicine platforms using different techniques and models, as has been shown for breast cancer and melanoma [122,130], is where the future of translational cancer research should point to for EC as well. In this view, initiatives like the recently established Models in Translational Oncology (MiTO) database will help to gather information about available models and help researchers in choosing the correct model to address a specific research question [131].

## Figures and Tables

**Table 1:** Endometrial cancer (EC) cell line information. Genomic alterations of the most commonly used type I and type II EC cell lines.

Cell Line	Tumor Location	Type	PTEN	KRAS	TP53	PI3K/Akt Pathways Alteration(s)	Microsatellite Instability
AN3CA	Metastasis	I	Deletion	wt	Missense mutation	Yes	High
ARK1	Primary	II	n/a	n/a	n/a	Yes	n/a
ARK2	Primary	II	n/a	n/a	n/a	n/a	n/a
ECC-1 ^1^	Primary	I	Missense mutation	wt	Missense mutation	Yes	High
HEC1A	Primary	I	wt	Missense mutation	Missense mutation	Yes	High
HEC1B	Primary	I	wt	Missense mutation	Missense mutation	Yes	Low
HEC50co	Metastasis	n/a	wt	Missense mutation	Deletion	n/a	n/a
Ishikawa	Primary	I	Deletion	wt	Missense mutation	Yes	High
KLE	Metastasis	n/a	wt	wt	Missense mutation	No	Low
MFE-280	Primary	I	wt	wt	Splice site mutation	Yes	Low
RL-95-2	Primary	I	Missense mutation	wt	Deletion	Yes	High
SPEC2	Primary	II	Not expressed	n/a	n/a	n/a	n/a

n/a, not available; ^1^ ECC-1 has been retracted from the market after the study by Korch et al. [23].

**Table 2:** Potential applications of EC cell lines for preclinical research.

Research Category	Field of Application	References
**Fundamental Research**	Molecular Biology	
	- Proliferation and migration	[23,25,29,36,37]
	- Tumorigenesis and dissemination mechanisms	
	- Therapy resistance mechanisms	
	- Pathways analysis and identification	
	Epigenetics	
	- DNA/histones modifications	[33,34,35,38,39,40,41,42,43,44]
	- Post-translational protein modification	
	- Non-coding RNAs	
	Metabolism	[45,46,47,48,49]
	- Hormone metabolism	
	- Glucose/glutamine metabolism	
	- Fatty acid metabolism	
	- Other	
	Functional analysis	[50]
	- New technologies development	
**Translational Research**	Drug discovery and validation	
	- Targeted therapies	
	- Overcoming therapy resistance	[25,30,36,49,51,52]
	Biomarkers discovery	
	- Distinguish different EC types	[36,43,53]
	- Identification of signatures linked to treatment response	

## Abbreviations

3D	Three-dimensional
Akt	Protein kinase B
AMPK	Adenosine monophosphate-activated protein kinase
ARID1A	AT-rich interaction domain 1A
BDII/Han	Berlin-Druckrey II/Hannover
CCLE	Cancer Cell Line Encyclopedia
CDK4,6	Cyclin-dependent kinase 4,6
Cdk6	Cyclin-dependent kinase 6
CMT1000	Center for Molecular Therapeutics 1000
Cre-lox	Cyclization recombinase-locus of X-over P1
CTNNB1	Catenin beta 1
DA/Han	Dark Agouti/Hannover
E:P	Estrogen:progesterone
EC	Endometrial cancer
EEC	Endometrioid endometrial cancer
EGFR	Epidermal growth factor receptor
ENNG	*N*-ethyl-*N*-nitro-*N*-nitrosoguanidine
ER	Estrogen receptor
ERBB2	Erb-b2 receptor tyrosine kinase 2
ERK1/2	Extracellular signal-regulated kinase 1/2
F344	Fischer 344
FBXW7	F-box and WD repeat domain containing protein 7
FGFR2	Fibroblast growth factor receptor 2
FIGO	International Federation of Gynecology and Obstetrics
Han:Wistar	Hannover Wistar
HOTAIR	Homeobox transcript antisense RNA
ICR	Institute of Cancer Research
JFCR-39	Japanese Foundation of Cancer Research 39
KRAS	KRAS proto-oncogene, GTPase
LKB1	Serine/threonine kinase 11
loxP	Locus of X-over P1
MDM4	Double minute 4 protein
MIG-6	Mitogen Inducible Gene 6
MLH	MutL homolog 1
MNU	*N*-methyl-*N*-nitrosourea
mTOR	Mammalian target of rapamycin
NCI60	National Cancer Institute 60
OOC	Organ-on-chip
PARP	Poly (ADP-ribose) polymerase
PI3K	Phosphoinositide 3-kinase
PIK3CA	Phosphatidylinositol-4,5-bisphosphate 3-kinase catalytic subunit alpha
PDX	Patient-derived xenograft
PDC	Patient-derived cell culture
PMS2	Mismatch repair endonuclease PMS2
POLE	Polymerase ε
POT1A	Protection of telomeres protein 1A
PR	Progesterone receptor
PTEN	Phosphatase and tensin homolog
STAT3STR	Signal transducer and activator of transcription 3Short tandem repeat
TP53	Tumor protein p53
Wnt	Wingless/integration-1
